# Contralateral grafts have comparable efficacy to ipsilateral grafts in anterior cruciate ligament reconstructions: a systematic review

**DOI:** 10.1186/s13018-023-04082-z

**Published:** 2023-08-11

**Authors:** DingYuan Fan, Jia Ma, Lei Zhang

**Affiliations:** 1 The First Department of Joint Surgery and Sports Medicine, Wangjing Hospital, Beijing, China; 2 Academy of Chinese Medical Sciences, No 6, South Zhonghuan Road, Chaoyang District, Beijing, 100102 People’s Republic of China; 3 Beijing University of Chinese Medicine, Beijing, China; 4 University College London, London, UK

**Keywords:** Contralateral, Ipsilateral, Anterior cruciate ligament, Anterior cruciate ligament reconstruction, Surgery, Knee, Arthroscopy

## Abstract

**Purpose:**

To perform a systematic review of the clinical outcomes of anterior cruciate ligament reconstruction using either contralateral or ipsilateral tendon autografts.

**Methods:**

A systematic review of literature published from inception to December 9, 2022, in multiple databases (PubMed, Embase, Scopus, and the Cochrane Library) was conducted in accordance with the 2020 PRISMA (Preferred Reporting Items for Systematic Reviews) guidelines. Two reviewers independently screened the literature, extracted the data, performed the risk of bias assessment and assessed the study quality. At least one of the following outcomes was evaluated for each study: muscle strength (isometric strength of the quadriceps or hamstring muscles, isokinetic peak flexion torque of the hamstring, or isokinetic peak extension torque of the hamstring), knee laxity examination, Lysholm score, pivot shift, International Knee Documentation Committee (IKDC) score, Knee Injury and Osteoarthritis Outcome Score (KOOS), Lachman test result, return to sports time, or incidence of complications. A random effects model was used for all analyses.

**Results:**

Four hundred scientific manuscripts were recovered in the initial search. After screening, 12 studies (2 randomized controlled trials, 9 cohort studies, and 1 case- control study) met the search criteria for the qualitative analysis. Among them, 9 cohort studies were used for the quantitative analysis. The results showed few statistically significant differences in terms of muscle strength (contralateral group versus ipsilateral group or donor site group versus ipsilateral group or donor site group versus nonoperative group), Lysholm score, and return to sports time. A comparison showed no significant differences in knee laxity, IKDC score, Tegner activity score, Lachman test score, or incidence of complication, or contralateral rupture.

**Conclusions:**

In anterior cruciate ligament reconstruction, the contralateral autologous tendon has a similar effect as the ipsilateral autologous tendon.

**Supplementary Information:**

The online version contains supplementary material available at 10.1186/s13018-023-04082-z.

## Introduction

Anterior cruciate ligament (ACL) tear is a sports-related injury that occurs in young, active individuals, and the annual incidence is increasing in many countries [1–4]. Because the ACL has little biological healing capacity after injury, anterior cruciate ligament reconstruction (ACLR) has become the gold standard for regaining stability, preventing early degeneration of the knee joint, and improving knee function [5, 6]. Graft selection is an important step affecting the prognosis of ACLR, and an ideal graft is associated with good postoperative rehabilitation, return to a full sporting function, and few complications [7, 8]. Current options include autografts, allografts, and artificial grafts [6, 9]. However, there is no consensus on the best graft for ACLR [8].

The advantages of the autologous tendon include no immune responses, faster graft incorporation, a high level of satisfaction, a lower level of laxity, and cost-effective [10–16]. However, during ACLR, the acquisition of the graft is usually from the injured limb on the same side. This is undoubtedly another heavy blow to the injured limb which may affect the patient's recovery process after surgery. Obtaining the graft from the contralateral limb can reduce the injury of the same limb allowing the injured limb to focus on ligamentation of the graft and provide favorable conditions for the rehabilitation of patients. If the rehabilitation process after surgery is shorter in patients with contralateral grafts than in patients with ipsilateral grafts, or if the sporting needs of the patient, especially the athlete, are met more quickly, the postoperative cost of ACL surgery will be much shorter and the injured athlete will be able to return to play as soon as possible. However, at present, the views of this technology are still debated.

The purpose of this systematic review was to collect the current clinical literature to assess the clinical and functional outcomes of contralateral autograft. We hypothesized that contralateral grafts and ipsilateral grafts have comparable clinical and functional outcomes in terms of ACLR.

## Materials and methods

### Review protocol

This systematic review was conducted in accordance with the 2020 Preferred Reporting Items for Systematic Reviews (PRISMA) guidelines (CRD42022342919) [17].

### Search strategy and selection criteria

Two reviewers independently searched Scopus, PubMed, the Cochrane Library, and Embase from database inception to the last research check on May 15, 2023. We searched the four databases using the following terms: (Ipsilateral contralateral) AND (((Anterior cruciate ligament) OR (Anterior cruciate ligament reconstruction)) OR (ACL)). Only studies available in the English language were included. Age was not a limitation for the search.

### Eligibility criteria

Studies were included if they met the following criteria:

- Type of participants: Patient of any age undergoing ACLR
- Intervention: Reconstruction only using an ipsilateral autogenous tendon.
- Comparator: Reconstructions only using the contralateral autogenous tendon.
- Outcome evaluation of at least one of the following: muscle strength (isometric strength of the quadriceps or hamstring muscles, isokinetic peak flexion torque of the hamstring, or isokinetic peak extension torque of the hamstring), knee anteroposterior laxity, Lysholm score, pivot shift, International Knee Documentation Committee (IKDC) score, Knee Injury and Osteoarthritis Outcome Score (KOOS), return to sport time, Lachman test result, or incidence of complications (including infection, patellar tendon re-rupture, and patellar fracture).

For patients with ipsilateral tendons, outcomes can be reported for the operated and non-operated limbs.

For patients with contralateral tendons, the outcome can be reported for the limb of the reconstructed surgical side and the tendon donor side.

- Average follow-up duration is at least 4 months.
- Study type: randomized controlled trial, prospective cohort study, retrospective cohort study, case‒control study.

### Exclusion criteria

1. Systematic review or review article
2. Laboratory study
3. Only reported anterior cruciate reconstruction with contralateral tendon grafts
4. Cross-sectional study
5. Studies with a partial overlap of patients that included in other studies published by the same author and outcome measures that without specific or sufficient data.
6. Case reports and case series
7. Two different types of tendons were used in the control and control groups

Two reviewers independently screened the studies recovered in the preliminary search by reading the title and abstract of the study. Irrelevant studies were excluded. Studies were further screened to confirm their relevance to the study and ensure that they met the final criteria. The third author resolved any disagreements during the selection process.

### Data extraction process

Two authors independently extracted the data. A standardized data extraction form was used to extract data from eligible studies. Any disagreements between the authors were resolved by discussion; if the dispute was not resolved, a third researcher was consulted. The mean value with standard deviation (SD) was the preferred extraction object; if not, the median, quartile range, and range (minimum–maximum) were extracted and converted during statistical analysis. The details of data extraction are shown in Appendix.

### Statistical analysis

Due to the heterogeneity and methodological design of the literature included in this study, the results are not summarized but presented as a narrative summary. Forest plots were graphed to display the collected outcome data for comparison. The mean differences were calculated for continuous variables along with 95% confidence intervals (95% CI). The risk ratio (RR) along with the 95% CI was calculated for dichotomous variables. All means, proportions, and relative risks of included studies are shown as a range of all values reported within the individual studies. A random effect model was applied for all results owing to the inherent heterogeneity expected in clinical studies. Data reported as the median, quartile range, or range were ultimately expressed as the mean ± SD using the Box‒Cox method as described by McGrath et al. [18]. When the same patients were evaluated at different follow-up times in two studies, we only included the most recently published study. Forest plots were performed using the standard software Review Manager Version 5.4

### Risk of bias assessment

For RCTs, the Cochrane risk of bias tool was applied, which includes the following items: sequence generation, allocation concealment, blinding, incomplete outcome data, selective reporting, and other biases [19]. Each item was graded as having a high risk, low risk, or unclear risk of bias [19]. For nonrandomized controlled studies (cohort and case‒control designs), the Newcastle‒Ottawa Scale (NOS) was used [20]. This instrument was used to evaluate the risk of bias based on three domains: selection, comparability, and outcomes [20]. A star system was used to classify the study quality, when a study met the criteria, it received a star from each item [20].

### Quality assessment

The methodological quality of each study was assessed with the Modified Coleman Methodology Score (MCMS), which comprises a 10-criterion validated score by two reviewers [21]. A score ranging from 85 to 100 was considered excellent, a score ranging from 70 to 84 was considered good, a score ranging from 55 to 69 was considered fair, and a score less than or equal to 54 was considered poor.

## Results

### Results of literature search and study selection

**Fig. 1.**
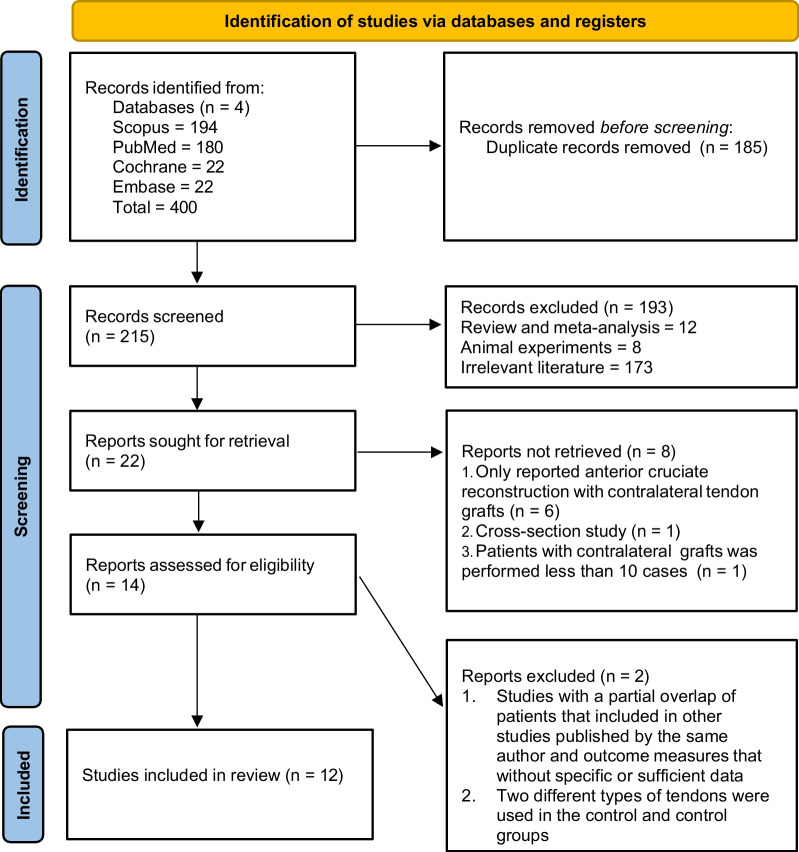
2020 PRISMA (Preferred Reporting Items for Systematic Reviews) flow diagram showing the literature search results, screening, and review

The search in the literature databases yielded 400 articles (180 PubMed, 4 Embase, 22 Cochrane, 194 Scopus) and after duplicates were excluded, 215 articles remained. Twenty-two articles were retrieved after screening the titles and abstracts. Unqualified studies were excluded, and 14 full-text articles were evaluated for further eligibility. Finally, a total of 12 articles [22–33] with 1762 patients were included in this study (Fig. 1).

### Study characteristics

There were 2 randomized controlled trials [26, 32] 9 cohort studies [22–33], and 1 case‒control study [31] that met the inclusion criteria. There were 2 articles [22, 31] from Japan, 2 articles [24, 27] from the USA, 2 articles [26, 33] from Canada, and 3 articles from Sweden [23, 29, 30]. In 2 studies [26, 33], researchers from Canada reported the same patients at different follow-up times. In 2 studies, researchers compared the ipsilateral versus contralateral limb results [29] and the donor versus non-operated limb results [30] in the same patients. All studies had at least a 4-month minimum follow-up time. Only one study [32] included males in the contralateral and ipsilateral groups (Table 1).

**Table 1:** Characteristics and details of the articles included in the systematic review

First author	Journal	Year	Country	Study design	LOE	Follow-up, mo	No. of patients	Age, y	Male/female sex, n
Yasuda et al	The American Journal of Sports Medicine	1995	Japan	PCS	2	24	IG:31 CG:34	IG:24 ± 7.4^a^ CG: 27 ± 8.4^a^	IG:18/13 CG:17/17
Kartus et al	The American Journal of Sports Medicine	1997	Sweden	RCS	3	IG:26 (20–33)^b^ CG:24 (22–30)^b^	IG:12 CG:12	IG:27 (23,33)^b^ CG:27 (24,33)^b^	IG:5/7 CG:5/7
Shelbourne et al	The American Journal of Sports Medicine	2000	the United States of America	RCS	3	24	IG:228 CG:434	IG:25.9 ± 9.0^a^ CG:23.9 ± 8.7^a^	IG:140/88 CG:267/167
Mastrokalos et al	The American Journal of Sports Medicine	2005	Germany	PCS	2	IG:31.7 CG:44.5	IG:52 CG:48	IG:35.4 (19–57)^b^ CG:35.9 (18–59)^b^	IG:32/20 CG:36/12
McRae et al	The American Journal of Sports Medicine	2013	Canada	RCT	1	24	IG:45 CG:50	IG:29.0 ± 9.4^a^ CG:29.5 ± 8.2^a^	IG:32/13 CG:28/22
Shelbourne et al	The American Journal of Sports Medicine	2014	the United States of America	RCS	3	24	IG:58 CG:279	IG:24.8 ± 9.5^a^ CG: 23.2 ± 8.9^a^	IG:20/38 CG:137/142
Legnani et al	European Journal of Orthopedic Surgery & Traumatology	2017	Italy	RCS	3	75.6 (24–108)^b^	IG:22 CG:23	IG:27.1 ± 9.8^a^ CG:26.8 ± 8.8^a^	IG:16/6 CG:14/9
Von Essen et al	Knee surgery, sports traumatology, arthroscopy	2021	Sweden	PCS	2	24	IG:68 CG:69	IG: 33 ± 9^a^ CG:31.1 ± 9^a^	IG:35/33 CG:44/25
Von Essen et al	Knee surgery, sports traumatology, arthroscopy	2021	Sweden	PCS	2	24	NG:64 DG:65	NG:33 ± 9^a^ DG:31.1 ± 9^a^	NG:33/31 DG: 42/23
Sanada et al	Journal of Experimental Orthopedics	2021	Japan	CCS	3	IG:20.9 CG:14.9	IG:15 CG: 15	IG:19.7 (14–27)^b^ CG:20.2 (16–36)^b^	IG:12/3 CG:11/4
De Souza Borges et al	The Knee	2022	Brazil	RCT	1	4	IG:44 CG:44	IG:26.3 ± 6.2^a^ CG:27.9 ± 8.9^a^	IG:44 M CG:44 M
Beaudoin A et al	Knee surgery, sports traumatology, arthroscopy	2022	Canada	PCS	2	151.2 ± 16.8	IG:23 CG:27	IG:41.9 ± 11.6^a^ CG:40.9 ± 7.5^a^	IG:15/8 CG:16/11

CG: contralateral group; DG: donor site group; IG: ipsilateral group; NG: nonoperative group; NA: not available; M: male

CCS: case‒control study; PCS: prospective cohort study; RCT: randomized controlled trial; RCS: retrospective cohort study

^a^Mean ± SD

^b^Mean with range

### Surgery detail

Patients with anterior cruciate ligament injuries received arthroscopic treatment in 10 studies. Bone-patellar tendon-bone grafts were used in 7 articles [22–25, 27, 31, 32], and the hamstring tendon was used in 5 studies [26, 28–30, 33]. Primary surgery was performed in 7 studies [24–27, 31–33], and revision surgery was performed in 2 studies [23, 28]. Postoperative rehabilitation was reported in all the studies except one [33] (Table 2).

**Table 2:** Summary of administered injections

First author	Surgery type	Harvest type	Primary/revision	Postoperative rehabilitation
Yasuda et al	Open with arthroscopy assist	Patellar tendon graft	NA	Yes
Kartus et al	Arthroscopy	Patellar tendon graft	Revision	Yes
Shelbourne et al	Open	Patellar tendon graft	Primary	Yes
Mastrokalos et al	Arthroscopy	Patellar tendon graft	Primary	Yes
McRae et al	Arthroscopy	Hamstring graft	Primary	Yes
Shelbourne et al	Open	Patellar tendon graft	Primary	Yes
Legnani et al	Arthroscopy	Hamstring graft	Revision	Yes
Von Essen et al	Arthroscopy	Hamstring graft	NA	Yes
Von Essen et al	Arthroscopy	Hamstring graft	NA	Yes
Sanada et al	Arthroscopy	Patellar tendon graft	Primary	Yes
De Souza Borges et al	Arthroscopy	Patellar tendon graft	Primary	Yes
Beaudoin A et al	Arthroscopy	Hamstring graft	Primary	No

IG: Ipsilateral Group; CG: contralateral Group; NA: not available

### Risk of bias assessment

Two RCTs [26, 32] had a high risk of blinding of participants and personnel, and one study had an unclear risk of blinding of outcome assessment (Table 3). Among the nonrandomized controlled studies, nine studies [22–25, 27–30, 33] showed good performance in selection, comparability, and outcomes (Table 4).

**Table 3:** Cochrane risk of bias assessment in randomized controlled studies

Study	Cochrane risk of bias tool						
	Random sequence generation	Allocation concealment	Blinding of participants and personnel	Blinding of outcome assessment	Incomplete outcome data	Selective reporting	Other sources of bias
McRae et al	Low	Low	High	Unclear	Low	Low	Low
De Souza Borges et al	Low	Low	High	Low	Low	Low	Low

**Table 4:** Newcastle–Ottawa scale (NOS) for assessing risk of bias in nonrandomized controlled studies

Assessment	Items	Yasuda et al	Kartus et al	Shelbourne et al	Mastrokalos et al	Shelbourne et al	Legnani et al	Von Essen et al	Von Essen et al	Sanada et al	Beaudoin A et al
Selection	1	★	★	★	★	★	★	★	★	★	★
	2	★	★	★	★	★	★	★	★	-	★
	3	★	★	★	★	★	★	★	★	-	★
Comparability	4	★	★	★	★	★	★	★	★	★	★
	5	★	★	★	★	★	★	★	★	★	★
	6	★	★	★	★	★	★	★	★	★	★
Outcome	7	★	★	★	★	★	★	★	★	★	★
	8	★	★	★	★	★	★	★	★	★	★
Total score		8	8	8	8	8	8	8	8	6	8

### Quality assessment

Two studies had a low score in terms of study sample size. [23, 31] In 7 studies [23–28, 33], researchers failed to obtain scores for the description of the technique used in ACLR. In 1 study [33], researchers failed to obtain a score for the description of the surgical procedure and postoperative rehabilitation (Table 5).

**Table 5:** Modified Coleman methodology score (MCMS) for assessing methodological quality in all studies

Assessment items		Yasuda et al	Kartus et al	Shelbourne et al	Mastrokalos et al	McRae et al	Shelbourne et al	Legnani et al	Von Essen et al	Von Essen et al	Sanada et al	De Souza Borges et al	Beaudoin A et al
Part A	1	10	4	10	10	10	10	7	10	10	4	10	7
	2	2	5	5	5	2	2	5	2	2	2	0	5
	3	10	0	0	0	0	0	0	10	10	10	10	0
	4	10	0	0	0	15	0	0	10	10	0	15	15
	5	5	5	5	5	5	5	5	5	5	5	2	5
	6	5	5	3	3	3	3	3	3	0	3	5	0
	7	10	10	10	10	10	10	10	10	10	10	10	0
Part B	8	10	10	10	10	10	10	10	10	10	10	10	10
	9	15	15	15	15	15	15	15	15	15	15	15	15
	10	15	15	15	15	13	15	15	15	15	15	15	13
Total score		92	69	73	73	83	70	70	90	87	74	92	70

### Outcomes of muscle strength

All results are presented in Table 6 and Additional file 2–7: Appendix Figs. 1–6.

**Table 6:** Results of muscle strength

Outcomes of muscle strength		Number of literature included	Range	Appendix figure
Isometric strength of quadriceps muscles (contralateral group versus ipsilateral group)	1 month	2	− 5.00 to 8.00	Additional file 2: Appendix Fig. S1A
	2–3 months	2	− 1.00 to 11.00	
	5–6 months	2	− 6.00 to 14.00	
	> 12 months	3	0.00 to 11.00	
Isometric strength of quadriceps muscles (donor site group versus ipsilateral group)	1 month	2	6.00 to 45.00	Additional file 2: Fig. S1B
	2–3 months	2	5.00 to 38.00	
	5–6 months	2	4.00 to 29.00	
	> 12 months	3	9.00 to 23.00	
Isometric strength of flexion hamstring muscles (contralateral group versus ipsilateral group)	5–6 months	2	4.00 to 10.00	Additional file 3: Appendix Fig. S2A
	> 12 months	2	0.00 to 10.00	
Isometric strength of flexion hamstring muscles (donor site group versus ipsilateral group)	5–6 months	2	18.00 to 25.00	Additional file 3: Appendix Fig. S2B
	> 12 months	2	3.00 to 19.00	
Isometric strength of flexion hamstring muscles (donor site group versus nonoperative group)	5–6 months	2	− 7.50 to − 20.00	Additional file 3: Appendix Fig. S2C
	> 12 months	2	− 16.00 to − 1.70	
Isokinetic peak flexion torque of the hamstring (contralateral group versus ipsilateral group)	NA	3	0.00 to 10.97	Additional file 4: Appendix Fig. S3
Isokinetic peak flexion torque of hamstring (donor site group versus ipsilateral group)	NA	3	0.00 to 9.70	Additional file 5: Appendix Fig. S4
Isokinetic peak flexion torque of hamstring (donor site group versus nonoperative group)	NA	2	− 23.00 to − 6.30	Additional file 6: Appendix Fig. 5
Isokinetic peak extension torque of hamstring (contralateral group versus ipsilateral group)	NA	2	− 10.53 to 0.00	Additional file 7: Appendix Fig. 6

NA: NA: not available

### Knee anteroposterior laxity

**Fig. 2:**
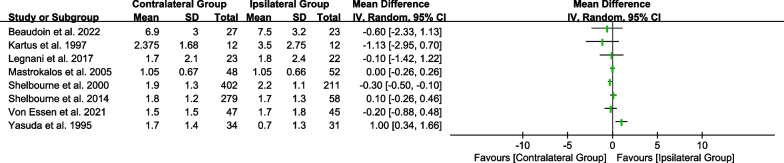
Forest plot showing knee anteroposterior laxity between the contralateral and ipsilateral groups. CI, confidence intervals; IV, inverse variance; SD, standard deviation

In nine studies [22–25, 27–29, 33], researchers compared anteroposterior laxity between the contralateral and ipsilateral groups. The results between the two groups ranged from -1.13 to 1.00 (Fig. 2).

### Lysholm score

**Fig. 3:**
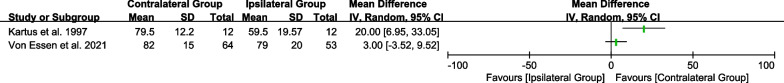
Forest plot showing the Lysholm score in the contralateral and ipsilateral groups. CI, confidence intervals; IV, inverse variance; SD, standard deviation

In two studies [23, 29], researchers reported the specific Lysholm scores in the contralateral and ipsilateral groups. One study [32] is presented as a graph without detailed data. The results between the two groups ranged from 3.00 to 20.00 (Fig. 3).

### IKDC

**Fig. 4:**
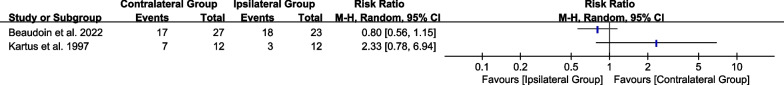
Forest plot showing the international knee documentation committee (IKDC) scores (presented as grade level) between in the contralateral and ipsilateral groups

In two studies [23, 33], researchers reported IKDC score as grade A or B between the contralateral and ipsilateral groups. The results between the two groups ranged from 0.80 to 2.33 (Fig. 4).

**Fig. 5:**
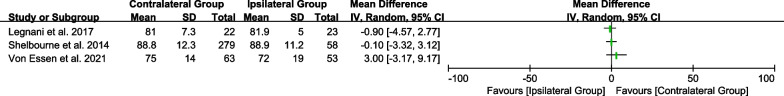
Forest plot showing the IKDC (presented as score) scores in the contralateral and ipsilateral groups. CI, confidence intervals; IV, inverse variance; M-H, Mantel‒Haenszel; SD, standard deviation

In three studies [27–29], researchers reported the IKDC scores of the contralateral and ipsilateral groups, and the results between the two groups ranged from − 0.90 to 3.00 (Fig. 5).

### Tegner activity score

**Fig. 6:**
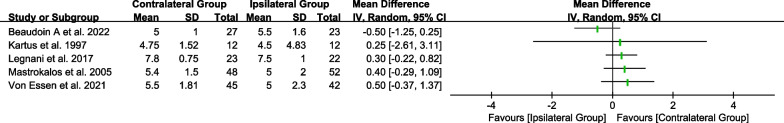
Forest plot showing the Tegner activity scores in the contralateral and ipsilateral groups. CI, confidence intervals; IV, inverse variance; M-H, Mantel‒Haenszel; SD, standard deviation

In five studies [23, 25, 28, 29, 33], researchers reported the Tegner activity scores of the contralateral and ipsilateral groups. The results between the two groups ranged from -0.50 to 0.50 (Fig. 6).

### KOOS

In two studies [28, 29], researchers reported the KOOS of the contralateral and ipsilateral groups. A forest plot could not be performed because one study [28] only showed the total score of KOOS, and there were no SD values with KOOS in one study [29].

### Lachman test

**Fig. 7:**
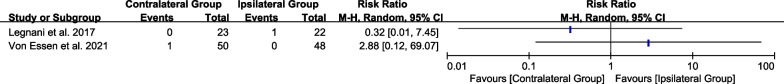
Forest plot showing the Lachman test results in the contralateral and ipsilateral groups. CI, confidence intervals; IV, inverse variance; M–H, Mantel‒Haenszel; SD, standard deviation

In two studies [28, 29], researchers reported the Lachman test results in the contralateral and ipsilateral groups. The results between the two groups ranged from 0.32 to 2.88 Lachman test positive incidence (Fig. 7).

### Return to sports time

**Fig. 8:**
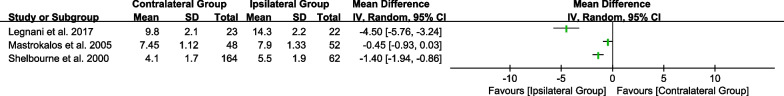
Forest plot of return to sports time between the contralateral and ipsilateral groups. CI, confidence intervals; IV, inverse variance; SD, standard deviation

In three studies [24, 25, 28], researchers reported the return to sports time in the contralateral and ipsilateral groups. The results between the two groups ranged from -4.50 to -0.45 months (Fig. 8).

### Contralateral rupture event

**Fig. 9:**
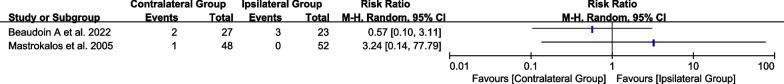
Forest plot showing the incidence of contralateral rupture in the contralateral and ipsilateral groups. CI, confidence intervals; IV, inverse variance; M-H, Mantel‒Haenszel

In two studies^,^ [25, 33] researchers reported the incidence of contralateral rupture in the contralateral and ipsilateral groups. The results between the two groups ranged from 0.57 to 3.24 contralateral rupture events (Fig. 9).

### Complications

**Fig. 10:**
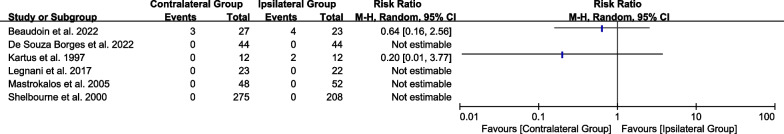
Forest plot showing the incidence of complications in the contralateral and ipsilateral groups. CI, confidence intervals; IV, inverse variance; M-H, Mantel‒Haenszel

In six studies [23–25, 28, 32, 33], researchers compared the incidence of complications. The results between the two groups ranged from 0.20 to 0.64 complication events (Fig. 10).

### Publication bias

**Fig. 11:**
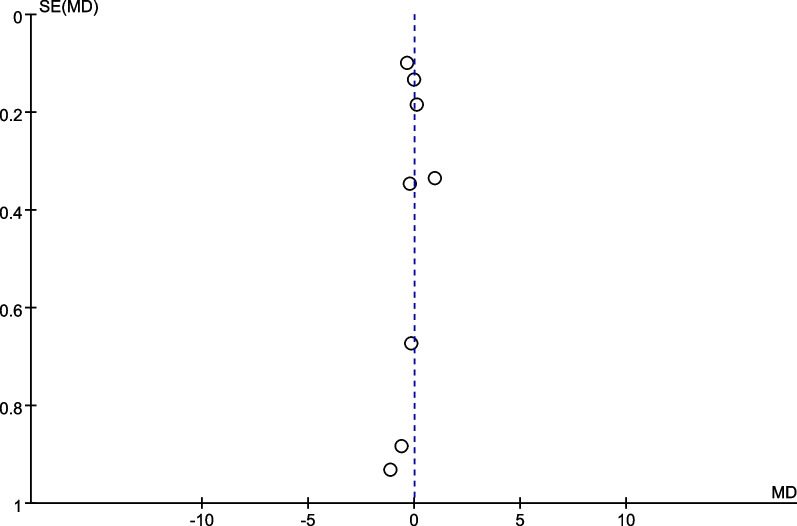
Funnel plot of knee anteroposterior laxity. MD, mean difference

Since eight studies [22–25, 27–29, 33] reported knee anteroposterior laxity data, the mean differences of knee anteroposterior laxity were plotted against the standard error in the funnel plots. The funnel plot showed some asymmetry, suggesting a publication bias for knee anteroposterior laxity (Fig. 11).

## Discussion

The most important finding of this study is that contralateral grafts and ipsilateral grafts for ACLR have equivalent results. The majority results showed similar clinical and functional outcomes.

### Outcomes of contralateral versus ipsilateral group

Primarily, the recovery of muscular strength is the goal of postoperative rehabilitation after a successful ACLR [34]. In our study, the results of the current review showed that the isometric strength of the quadriceps muscles (1 month, 2–3 months, and 5–6 months), the isometric strength of the flexion hamstring muscles (5–6 months, ≥ 12 months), the isokinetic peak flexion torque of the hamstring and the isokinetic peak extension torque of the hamstring were comparable. Notably, the isometric strength of the quadriceps muscles of the contralateral group was better than that of the ipsilateral group after 12 months. One reason for this result is that an additional article [27] in which the isometric strength of the quadriceps muscles at 1 month, 2–3 months, and 5–6 months was included. It indicates that the efficiency of the statistical results is insufficient. Although the outcome was not stable, it at least showed that the recovery of muscle strength after ACL reconstruction with the contralateral grafts was not inferior to that with the ipsilateral grafts. Abnormal knee laxity is often associated with unstable knees, meniscal injuries and early onset osteoarthritis after ACLR [35]. In this study, there were no significant differences in knee laxity between the two groups and it shows that the method of obtaining contralateral graft is reliable from the perspective of postoperative knee recovery. In addition, the results of the Lachman test also showed similar results, which further demonstrated the credibility of the knee laxity results. The IKDC score is employed in the assessment of quality of life in terms of symptoms and disabilities relevant to patients with knee disorders [36]. The Tegner activity scale grades activity level based on work and sports activities after ACL and meniscal injuries [37]. The consistency of the three scores indicates that the contralateral graft technique can also achieve satisfactory results. The goal of ACLR is to help patients return to their preinjury level of movement [38]. Choosing to return to sport is still an important decision [39]. The results may show a shorter time to return to sport after surgery, but the current result is underpowered to draw reliable inferences from the available data. Contralateral ACL injury is one of the most devastating outcomes after ipsilateral ACLR [40]. It is worth considering that contralateral grafts cause additional damage to the donor limb when compared to ipsilateral grafts and may increase the risk of contralateral ACL injury when compared to ipsilateral grafts. However, in this study, the results showed no significant difference between contralateral and ipsilateral grafts. There were also no significant differences between the two groups in terms of complications, suggesting that the contralateral graft technique does not increase the risk of the procedure.

### Outcomes of donor site versus ipsilateral group

For the graft donor side of the limb, there was no significant difference in the isometric strength of the quadriceps muscles at 1 month, 2–3 months, and 5–6 months compared with the ipsilateral ACLR limb. This indicates that one of the main causes of limb muscle strength decline in the early stage is still grafting. Regarding the isometric strength of the quadriceps muscles after 12 months, the results indicated that the donor-side limb was preferred over the ipsilateral limb.

The results showed that ACLR became the main factor affecting the recovery of limb muscle strength in the later stage. The hamstring isometric strength of the flexor leg muscles was better in the donor limb at 5–6 months, but there was no significant difference after 12 months. This may be related to the gradual completion of ligamentalization of the graft, bone tunnel healing and limb adaptation. Due to insufficient data from each study, the result of the isokinetic peak torque flexion hamstring only indicated that there was no significant difference between the two groups at the final follow-up.

Compared with the ipsilateral autograft technique, the contralateral autograft technique reduces the risk of injury to the ipsilateral limb by transferring the graft harvest to the contralateral side. In theory, this creates a good environment for the rehabilitation of the ipsilateral limb, because trauma was divided between the two knees. The inflammation, damage and soft tissues swelling of the injured limb should be reduced [31, 41] However, in the early postoperative period, results showed no significant difference in muscle strength between the two techniques. This may be related to the simultaneous rehabilitation programs of both knees after the operation [32]. Another reason may be that the recovery of muscle strength after ACLR depends only on the difference between the two limbs, not on which limb the graft was taken from [31]. Although some patients may be concerned that having surgery on both limbs will affect their ability to engage in sports, current evidence shows that the contralateral graft technique has comparable clinical and functional outcomes as the ipsilateral graft technique. Contralateral grafts can be used as an alternative source of ipsilateral grafts.

In ACLR, there are three options, including allografts, and artificial grafts and autografts [6, 9]. Compared with the first two types of grafts, autologous tendons are removed from the patient's own body and therefore, do not cost extra for the grafts. Therefore, it is undoubtedly the first choice for low- and middle-income patients. In addition, autologous tendons do not produce an immune response [10–12], and it seems to be the only option for patients with immune problems when they suffer from ACL tear. However, when revision surgery for ACLR is required, it is cruel to obtain tendons from the same limb and this will be detrimental to the postoperative functional recovery of patients. Under these conditions, it is advisable to obtain the tendon from the opposite side.

### Strengths

Compared with a previous systematic review [42], this study also included studies with different autologous materials, such as hamstring tendons. We also included information about donor site limbs and nonoperative limbs. These advantages make the conclusion of our paper more comprehensive and convincing.

### Limitations

Most importantly, high-quality RCTs are still lacking, and the evidence strength of this study is low. As a result, we were unable to conduct meta-analysis to synthesize the results. Second, types of surgical technique, grafts and primary or revision surgery are inconsistent in the included literature, which may cause some heterogeneity in the results. However, in patients undergoing revision surgery, the type of graft (ipsilateral autologous tendon or allogeneic tendon or artificial ligament) used during the initial surgery may also affect the outcome. More importantly, some patients were lost due to the long follow-up time, which may have biased the results. Fourth, there is no comparison of the quadriceps tendon in ipsilateral versus contralateral ACLR in this article, which is also a limitation of the study. Fifth, there are few articles in which researchers report the specific condition of the donor side of the limb, which makes our results incomplete. There is also a lack of outcome measures with high sensitivity to evaluate. In addition, most researchers did not report whether the included patients played competitive sports, so it remains unclear whether ipsilateral versus contralateral tendon grafts have comparable outcomes in athletes undergoing ACLR.

## Conclusions

In ACLR, the contralateral autologous tendon has a similar effect as the ipsilateral autologous tendon.

### Supplementary Information

**Additional file 1.**

**Additional file 2: Appendix Fig. S1A.** Forest plot showing the isometric strength of the quadriceps muscles (contralateral group versus ipsilateral group). CI, confidence intervals; IV, inverse variance; SD, standard deviation. **Appendix Fig. 1B.** Forest plot showing the isometric strength of the quadriceps muscles. (Donor site group versus ipsilateral group). CI, confidence intervals; IV, inverse variance; SD, standard deviation.

**Additional file 3: Appendix Fig. S2A.** Forest plot showing the isometric strength of the flexion hamstring muscles (contralateral group versus ipsilateral group). CI, confidence intervals; IV, inverse variance; SD, standard deviation. **Appendix Fig. 2B.** Forest plot showing the isometric strength of the flexion hamstring muscles (donor site group versus ipsilateral group). CI, confidence intervals; IV, inverse variance; SD, standard deviation. **Appendix Fig. 2C.** Forest plot showing the isometric strength of the flexion hamstring muscles (donor site group versus nonoperative group). CI, confidence intervals; IV, inverse variance; SD, standard deviation.

**Additional file 4: Appendix Fig. S3.** Forest plot showing the isokinetic peak flexion torque of the hamstring (Contralateral group versus Ipsilateral group). CI, confidence intervals; IV, inverse variance; SD, standard deviation.

**Additional file 5: Appendix Fig. S4.** Forest plot showing the isokinetic peak flexion torque of the hamstring (donor site group versus ipsilateral group). CI, confidence intervals; IV, inverse variance; SD, standard deviation.

**Additional file 6: Appendix Fig. S5.** Isokinetic peak flexion torque of the hamstring (donor site group versus nonoperative group). CI, confidence intervals; IV, inverse variance; SD, standard deviation.

**Additional file 7: Appendix Fig. S6.** Isokinetic peak extension torque of the hamstring (contralateral group versus ipsilateral group). CI, confidence intervals; IV, inverse variance; SD, standard deviation.

## Data Availability

The present study was a review of the previously published literature.

## Abbreviations

ACL: Anterior cruciate ligament
ACLR: Anterior cruciate ligament reconstruction
CI: Confidence Intervals
LOE: Level of evidence
MCMS: Modified Coleman Methodology Score
NOS: Newcastle‒Ottawa Scale (NOS)
IKDC: International Knee Documentation Committee
KOOS: Knee Injury and Osteoarthritis Outcome Score
PRISMA: Preferred Reporting Items for Systematic Reviews
RR: Risk Ratio
SD: Standard Deviation
