# Supplementary material for: Contralateral grafts have comparable efficacy to ipsilateral grafts in anterior cruciate ligament reconstructions: a systematic review
Source: J Orthop Surg Res. 2023 Aug 11;18:596. doi: 10.1186/s13018-023-04082-z (PMC10422826; doi:10.1186/s13018-023-04082-z)
Supplement: Supplementary file 1 — Additional file 1. [file 13018_2023_4082_MOESM1_ESM.docx]

**The details of data extraction are as follows:**

•Study characteristics: first author, journal, year, country, study design, level of evidence (LOE), and follow-up (month).

•Population characteristics: number of patients, age (year), male/female (sex, n).

•Surgery type (open or arthroscopy)

•Harvest Type

•Primary Surgery or Revision Surgery

•Postoperative Rehabilitation

•Muscle strength:

1. Isometric Strength of the Quadriceps Muscle

Including the contralateral group, the ipsilateral group, the donor site group, and the nonoperative group at 1 month, 2-3 months, 5-6 months, and ≥12 months;

2. Isometric Strength of the Hamstring Muscles

Including contralateral group, ipsilateral group, donor site group, nonoperative group at 1 month, 2-3 months, 5-6 months, ≥12 months;

3. Isokinetic peak flexion torque of the Hamstring

The included groups were the contralateral group, the ipsilateral group, the donor site group, and the nonoperative group.

Data on the latest outcome measures were included;

4. Isokinetic peak extension torque of the Hamstring

The included groups were the contralateral group, the ipsilateral group, the donor site group, and the nonoperative group.

Data on the latest outcome measures were included;

•Knee anteroposterior laxity (mm)

•Lysholm score

•The international knee documentation committee (IKDC) questionnaire

On the form, patients were categorized as grades or according to score.

•Tegner activity score

•Lachman test result

•Return to sports time (months)

•Contralateral rupture

•Complication

Including infection, patellar tendon re-rupture, and patellar fracture.
