# Supplementary figures and images for: Contralateral grafts have comparable efficacy to ipsilateral grafts in anterior cruciate ligament reconstructions: a systematic review
Source: J Orthop Surg Res. 2023 Aug 11;18:596. doi: 10.1186/s13018-023-04082-z (PMC10422826; doi:10.1186/s13018-023-04082-z)

A

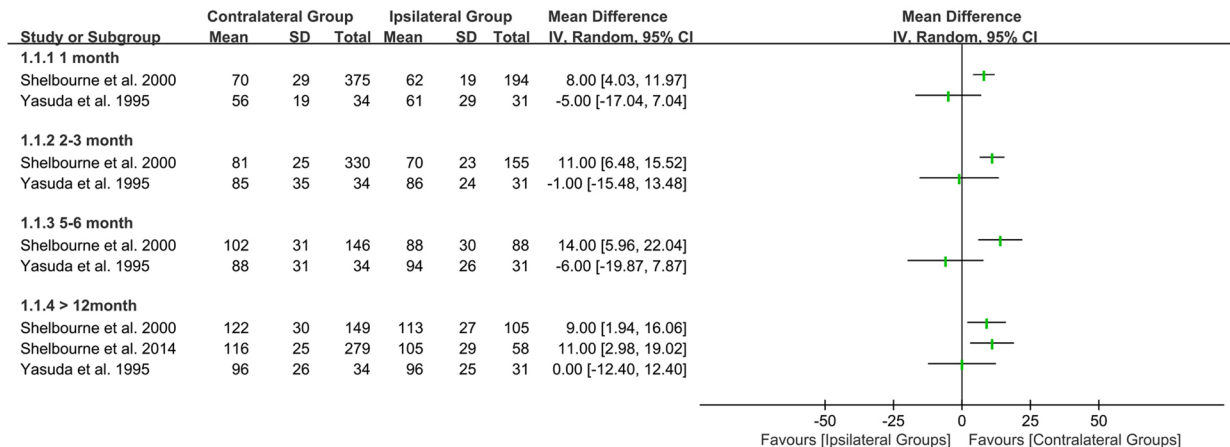

B

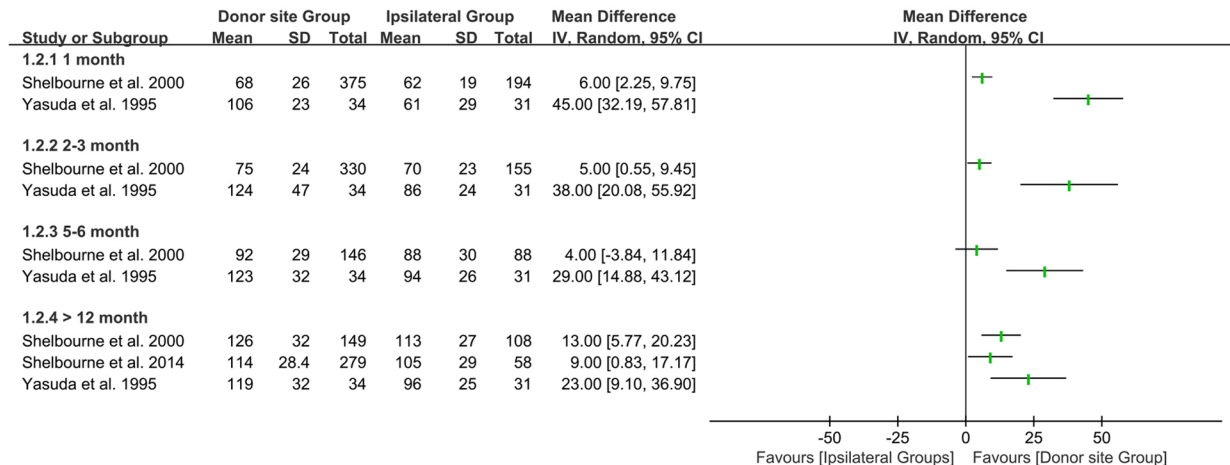

Supplement: Supplementary file 2 — Additional file 2: Appendix Fig. S1A. Forest plot showing the isometric strength of the quadriceps muscles (contralateral group versus ipsilateral group). CI, confidence intervals; IV, inverse variance; SD, standard deviation. Appendix Fig. 1B. Forest plot showing the isometric strength of the quadriceps muscles. (Donor site group versus ipsilateral group). CI, confidence intervals; IV, inverse variance; SD, standard deviation. [file 13018_2023_4082_MOESM2_ESM.pdf]

A

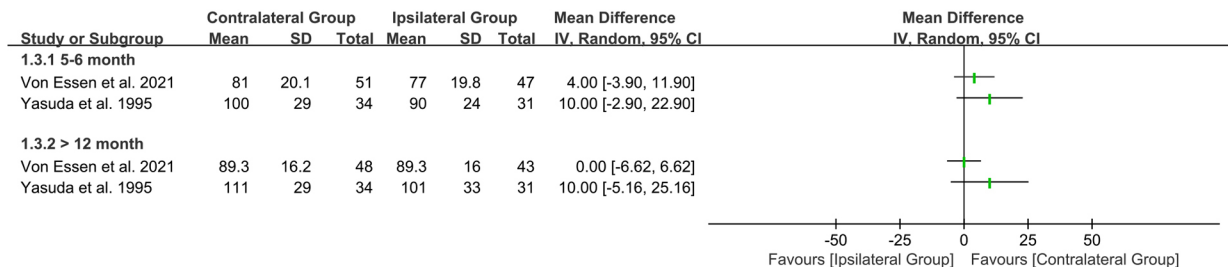

B

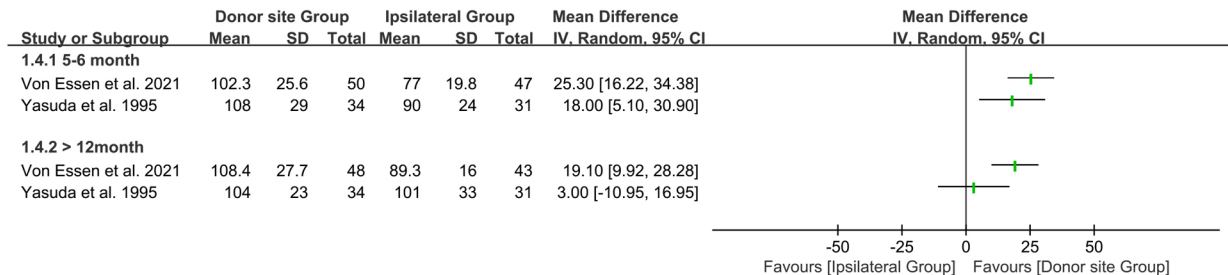

C

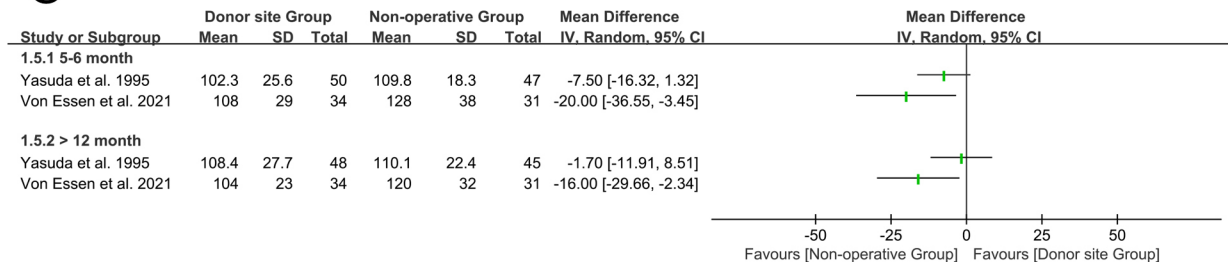

Supplement: Supplementary file 3 — Additional file 3: Appendix Fig. S2A. Forest plot showing the isometric strength of the flexion hamstring muscles (contralateral group versus ipsilateral group). CI, confidence intervals; IV, inverse variance; SD, standard deviation. Appendix Fig. 2B. Forest plot showing the isometric strength of the flexion hamstring muscles (donor site group versus ipsilateral group). CI, confidence intervals; IV, inverse variance; SD, standard deviation. Appendix Fig. 2C. Forest plot showing the isometric strength of the flexion hamstring muscles (donor site group versus nonoperative group). CI, confidence intervals; IV, inverse variance; SD, standard deviation. [file 13018_2023_4082_MOESM3_ESM.pdf]

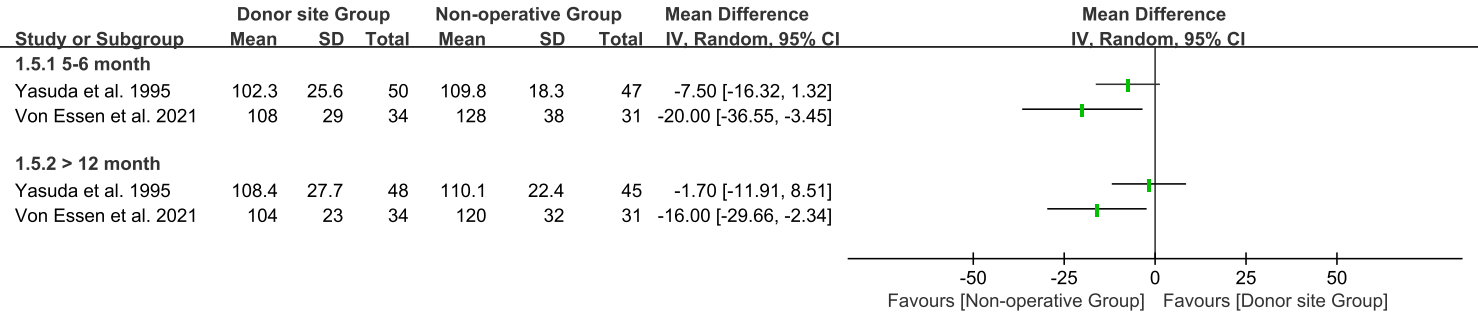

Supplement: Supplementary file 4 — Additional file 4: Appendix Fig. S3. Forest plot showing the isokinetic peak flexion torque of the hamstring (Contralateral group versus Ipsilateral group). CI, confidence intervals; IV, inverse variance; SD, standard deviation. [file 13018_2023_4082_MOESM4_ESM.pdf]

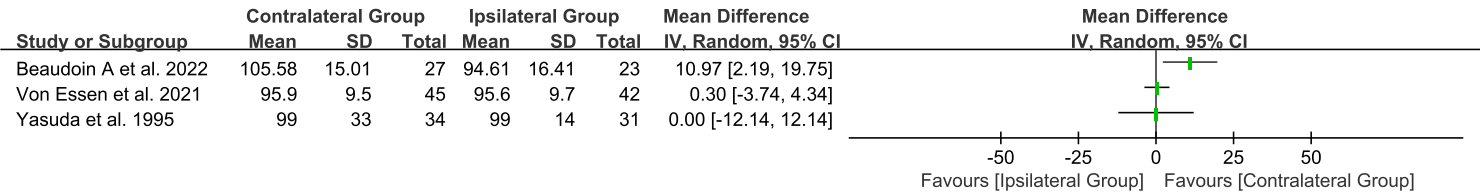

Supplement: Supplementary file 5 — Additional file 5: Appendix Fig. S4. Forest plot showing the isokinetic peak flexion torque of the hamstring (donor site group versus ipsilateral group). CI, confidence intervals; IV, inverse variance; SD, standard deviation. [file 13018_2023_4082_MOESM5_ESM.pdf]

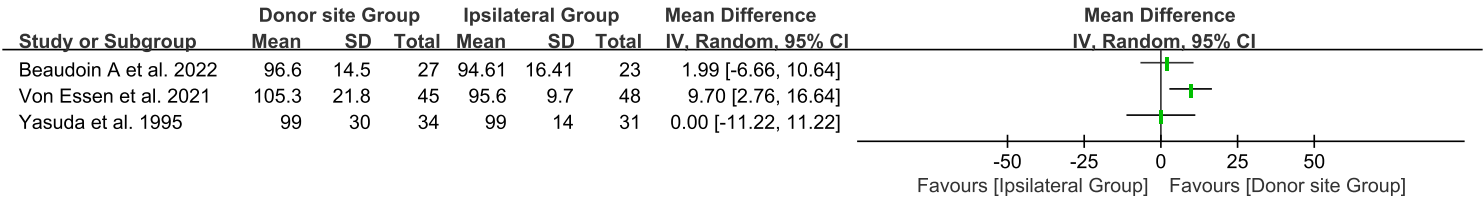

Supplement: Supplementary file 6 — Additional file 6: Appendix Fig. S5. Isokinetic peak flexion torque of the hamstring (donor site group versus nonoperative group). CI, confidence intervals; IV, inverse variance; SD, standard deviation. [file 13018_2023_4082_MOESM6_ESM.pdf]

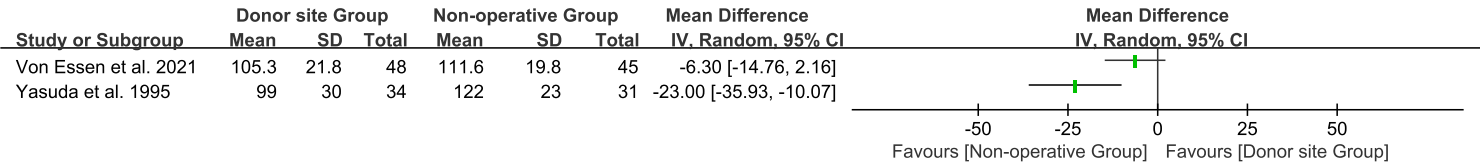

Supplement: Supplementary file 7 — Additional file 7: Appendix Fig. S6. Isokinetic peak extension torque of the hamstring (contralateral group versus ipsilateral group). CI, confidence intervals; IV, inverse variance; SD, standard deviation. [file 13018_2023_4082_MOESM7_ESM.pdf]
